# Treadmill Exercise Prevents Cognitive Impairments in Adolescent Intermittent Ethanol Rats by Reducing the Excessive Activation of Microglia Cell in the Hippocampus

**DOI:** 10.3390/ijms232314701

**Published:** 2022-11-25

**Authors:** Yanxia Guo, Min Yan, Li Li, Li Zhao, Yan Li

**Affiliations:** 1 Department of Exercise Physiology, Beijing Sport University, Beijing 100084, China; 2 Key Laboratory of Physical Fitness and Exercise, Ministry of Education, Beijing Sport University, Beijing 100084, China

**Keywords:** adolescent intermittent ethanol, neuroinflammation, microglia cell, endocannabinoid system, exercise

## Abstract

The excessive activation of microglia cell induced by adolescent intermittent ethanol (AIE) leads to neuroinflammation in the hippocampus. The endocannabinoid system plays a key role in the modulation of microglia activation. Accumulating evidence suggests that regular exercise improves learning and memory deficits in AIE models. The purpose of this study was to explore the effects of treadmill exercise intervention on the cognitive performance, activation of microglia cells and the expression of monoacylglycerol lipase (MAGL), cannabinoid receptor type 1 (CB1R) and cannabinoid receptor type 2 (CB2R) in the hippocampus of AIE rats. Here, we show that AIE rats exhibited cognitive impairments, whereas the treadmill exercise improves the cognitive performance in AIE rats. In order to explore the possible mechanisms for the exercise-induced attenuation of cognitive disorder, we examined the neuroinflammation in the hippocampus. We found that treadmill exercise led to the decrease in the level of proinflammatory cytokines (IL-1β, IL-6 and TNF-α) and the increase in the level of anti-inflammatory cytokine (IL-10). In addition, we found that treadmill exercise reduced the excessive activation of the microglia cell in the hippocampus of AIE rats. Finally, we found that AIE led to a decrease in the expression of CB1R and CB2R in the hippocampus; however, the treadmill exercise further decreased the expression of CB2R in the hippocampus of AIE rats. Our results suggest that treadmill exercise attenuates AIE-induced neuroinflammation and the excessive activation of hippocampus microglial cells, which may contribute to the exercise-induced improvement of cognitive performance in AIE rats.

## 1. Introduction

Alcohol abuse is a growing public health problem worldwide [1,2]. In comparison to adults, adolescents are more likely to feel the stimulant effects of alcohol and exhibit poorer self-control, which results in the widespread binge drinking of alcohol among adolescents [3]. According to the Global Status Report on Alcohol and Health 2018, more than a quarter (26.5%) of all 15–19-year-olds are current drinkers, amounting to 155 million adolescents [4]. Adolescence represents an important period for the development of the brain, and alcohol intake during this period can disturb adolescent brain development and functioning [5,6,7]. It has been observed that the binge drinking of alcohol in adolescence leads to the dysfunction of synaptic transmission efficacy and synaptic plasticity, and it is believed that these impairments play a key role in persistent cognitive and psychiatric abnormalities during adulthood [8,9,10].

The hippocampus is an important brain area that processes cognition and emotion. Chronic exposure to alcohol causes neuroinflammation in the hippocampus resulting from increased proinflammatory cytokine production and microglial cell activation [11,12,13,14]. Accumulating evidence suggests that neuroinflammation in the hippocampus contributes to the impairment of synaptic transmission efficacy, which is important for cognitive performance [15,16]. In support of this idea, a recent study indicated that the binge drinking of alcohol in adolescence increases the pathological processes of early Alzheimer’s disease during adulthood through the enhancement of neuroimmune activation [17]. In animal studies, adolescent intermittent ethanol (AIE) exposure would increase the release of pro-inflammatory factors, decrease the release of anti-inflammatory factors and increase the activation of microglia [13]. Additionally, anti-inflammatory treatments can attenuate the AIE-induced neurological disorders in animal models [16,18,19]. However, the activation of microglia was not observed in some chronic alcohol exposure patients using positron emission tomography (PET) [20]. Nevertheless, this result was observed in alcohol-dependent patients within 1 month of medically assisted withdrawal, but not in adolescent binge drinkers. In addition to the activation of microglia, astrocyte can also be activated by alcohol exposure [21,22], whereas when pathological damage occurs, microglia is activated faster than astrocyte and modulates the activation of astrocyte [23]. Thus, the present study will examine the effects of AIE on the neuroinflammation and activation of microglia in the hippocampus of rats.

Accumulating evidence suggests that regular exercise intervention delays the process of pathological changes and improves learning and memory deficits in AIE models [14,24,25,26]. Mechanisms underlying exercise-induced protection on cognitive performance against AIE have also been explored. Exercise modulates the expressions of proteins regulating synaptic transmission and synaptic plasticity [25,27,28]. Additionally, exercise restores the loss of neurogenesis in the brain of AIE models [16,24,29]. Furthermore, exercise reverses AIE-induced deficits in dendritic morphology [26]. In previous studies, it has been shown that exercise blunts AIE-induced neuroimmune activation in the dorsal raphe nucleus and basal forebrain [25,29]. However, it remains unknown whether exercise affects the AIE-induced excessive microglia activation and neuroinflammation in the hippocampus. In the present study, we determined whether exercise attenuated the AIE-induced dysfunction of cognitive performance in early adulthood. Additionally, we explored whether exercise altered AIE-induced excessive microglia activation and neuroinflammation in the hippocampus in early adulthood. It has been shown that the endocannabinoid system (ECS) plays a significant role in mediating neuroinflammatory responses [30]. AIE-induced changes in the ECS contributes to behavioral alterations. It has been shown that AIE decreased the expression of cannabinoid receptor type 1 (CB1R), increased the expression of monoacylglycerol lipase (MAGL) and disrupted a form of excitatory long-term depression that is dependent on CB1R [8]. Furthermore, preventing CB1R activation could be a beneficial strategy to avoid alcohol-induced brain damage [30]. These results suggest that ECS might be a potential target in the treatment of AIE-induced cognitive impairment. However, it remains unknown whether exercise affects the ECS in the hippocampus of AIE models. In the present study, we explored the effects of treadmill exercise intervention on the expression of MAGL, CB1R and cannabinoid receptor type 2 (CB2R) in the hippocampus of AIE rats. We found that exercise improved the cognitive performance of AIE rats during early adulthood. Exercise decreased the level of proinflammatory cytokines and increased the level of anti-inflammatory cytokines, leading to a reduction in the neuroinflammation in the hippocampus of AIE rats. In addition, exercise led to a decrease in the activation of microglia in the hippocampus of AIE-rats. Furthermore, AIE decreased the expressions of CB1R and CB2R in the hippocampus of AIE rats, while exercise decreased the CB2R in AIE rats. These results reveal a potential mechanism for exercise-induced protection against AIE-related cognitive decline during early adulthood.

## 2. Results

### 2.1. Treadmill Exercise Prevented AIE-Induced Decline in Spatial Learning and Memory in Young Adult Rats

**Figure 1:**
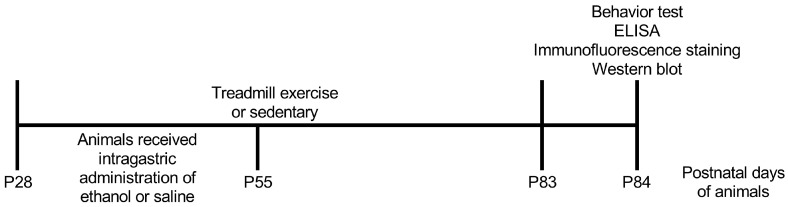
Timeline of the experiment.

**Figure 2:**
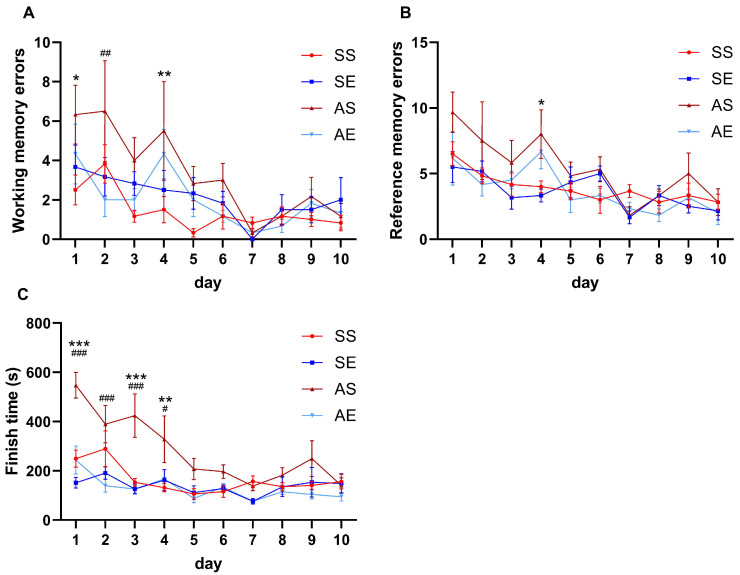
Treadmill exercise attenuated AIE-induced cognitive impairment in spatial learning and memory in young adult rats. (**A**) The working memory errors of rats in each group. The working memory errors on day 1 and day 4 were significantly increased in the AS rats compared to the SS rats. The working memory errors on day 2 were significantly decreased in AE rats compared to AS rats (* *p* < 0.05, SS vs. AS; ** *p* < 0.01, SS vs. AS; ## *p* < 0.01, AS vs. AE; *n* = 6). (**B**) The reference memory errors of rats in each group. The reference memory errors on day 4 were significantly increased in the AS rats compared to the SS rats (* *p* < 0.05, SS vs. AS; *n* = 6). However, the treadmill exercise intervention had no effect on the reference memory errors in AE rats compared with the AS rats (*p* > 0.05). (**C**) The finish times of the rats in each group. The finish times on day 1, day 3 and day 4 were significantly increased in the AS rats compared to the SS rats. The finish times on day 1, day 2, day 3 and day 4 were significantly decreased in AE rats compared to AS rats (** *p* < 0.01, SS vs. AS; *** *p* < 0.001, SS vs. AS; # *p* < 0.05, AS vs. AE; ### *p* < 0.001, AS vs. AE; *n* = 6).

We first sought to explore whether AIE rats displayed cognitive dysfunction in spatial learning and memory and whether treadmill exercise ameliorated the reduction in cognitive performance in spatial learning and memory in young adult rats. All rats were assigned into four group: saline sedentary (SS) group, saline exercise (SE) group, AIE sedentary (AS) group and AIE exercise (AE) group. From postnatal day 28 (P28) to P55, rats received the AIE paradigm. From P28 to P55, rats received 8 weeks of exercise intervention or non-exercise control intervention (2 × 2 factor design: AIE vs. exercise). The timeline of the experiment is shown in Figure 1. After 8 weeks of exercise intervention, the eight-arm radial maze test was used to evaluate the cognitive performance in spatial learning and memory of rats. The working memory, the reference memory and the finish time were measured. As shown in Figure 2, the working memory error, reference memory error and finish time were all significantly decreased during the process of the test in rats of four groups (working memory errors, F = 5.12, *p* < 0.001, Figure 2A; reference memory errors, F = 7.35, *p* < 0.001, Figure 2B; finish time, F = 9.58, *p* < 0.001, Figure 2C). 

For working memory error, a repeated-measures ANOVA showed that the group had a significant main effect on the working memory error (F = 3.98, *p* = 0.029), time had a significant main effect on the working memory error (F = 5.12, *p* < 0.001), but there was no significant interaction between group and time on the working memory error (F = 1.17, *p* = 0.276). The multiple comparison test showed that the working memory error was significantly increased in the AS rats than those in the SS rats on day 1 and day 4 (day 1, *p* = 0.012; day 4, *p* = 0.008, Figure 2A). Meanwhile, exercise decreased the working memory error in the AIE rats on day 2 (*p* = 0.002, Figure 2A).

For reference memory error, a repeated-measures ANOVA showed that the group had a significant main effect on the reference memory error (F = 3.81, *p* = 0.033), time had a significant main effect on the reference memory error (F = 7.35, *p* < 0.001), but there was no significant interaction between group and time on the reference memory error (F = 1.01, *p* = 0.462). The multiple comparison test showed that the reference memory error was significantly increased in the AS rats than those in the SS rats on day 4 (*p* = 0.034, Figure 2B). Meanwhile, there was no significant effect of exercise on the reference memory error in the AIE rats (*p* > 0.05, Figure 2B).

For the finish time, a repeated-measures ANOVA showed that the group had a significant main effect on the finish time (F = 7.98, *p* = 0.002), time had a significant main effect on the finish time (F = 9.58, *p* < 0.001) and there was a significant interaction between the group and time at the finish time (F = 3.28, *p* < 0.001). A multiple comparison test showed that the finish time was significantly increased in the AS rats than those in the SS rats on day 1, day 3 and day 4 (day 1, *p* < 0.001; day 3, *p* < 0.001; day 4, *p* = 0.005; Figure 2C). Meanwhile, the exercise decreased the finish time in the AIE rats on day 1–day 4 (day 1, *p* < 0.001; day 2, *p* < 0.001; day 3, *p* < 0.001; day 4, *p* = 0.030; Figure 2C).

Together, these findings suggested that AIE led to impairments in cognitive performance in rats. An eight-week treadmill exercise intervention restored the cognitive performance in AIE rats.

### 2.2. Treadmill Exercise Reversed AIE-Induced Inflammation in the Hippocampus of Young Adult Rats

**Figure 3:**
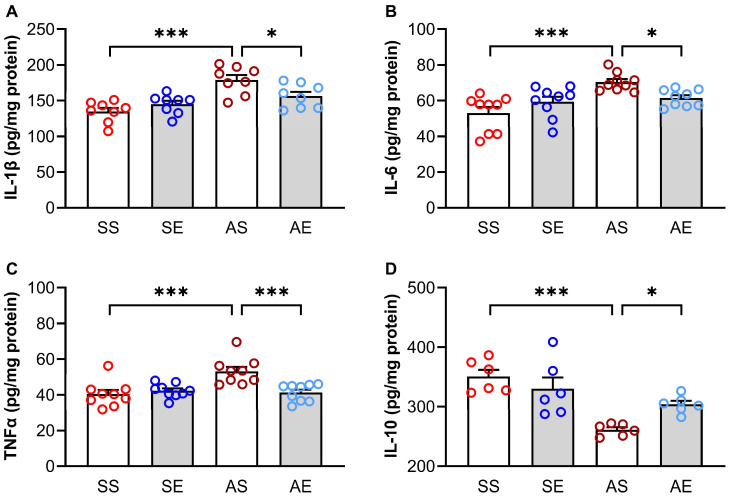
Treadmill exercise reversed AIE-induced inflammation in the hippocampus of young adult rats. (**A**) The protein level of IL-1β in the hippocampus of rats in each group. The level of IL-1β was increased in the AS rats compared to the SS rats. The increased level of IL-1β was prevented by treadmill exercise intervention (* *p* < 0.05, *** *p*< 0.001, *n* = 8). (**B**) The protein level of IL-6 in the hippocampus of rats in each group. The level of IL-6 was increased in the AS rats compared to the SS rats. The increased level of IL-6 was prevented by treadmill exercise intervention (* *p* < 0.05, *** *p* < 0.001, *n* = 9). (**C**) The protein level of TNF-α in the hippocampus of rats in each group. The level of TNF-α was increased in the AS rats compared to the SS rats. The increased level of TNF-α was prevented by treadmill exercise intervention (*** *p* < 0.001, *n* = 9). (**D**) The protein level of IL-10 in the hippocampus of rats in each group. The level of IL-10 was decreased in the AS rats compared to the SS rats. The decreased level of IL-10 was prevented by treadmill exercise intervention (* *p* < 0.05, *** *p* < 0.001, *n* = 6).

AIE-induced neuroinflammation in the hippocampus contributes to cognitive dysfunction. To investigate whether treadmill exercise-induced enhancement in cognitive performance was associated with the modulation of AIE-induced neuroinflammation, we detected the protein levels of proinflammatory factors (IL-1β, IL-6 and TNF-α) and anti-inflammatory factor (IL-10). A two-way ANOVA showed that AIE and exercise had significant effects on the protein levels of IL-1β (AIE, F = 23.46, *p* < 0.001; exercise, F = 1.16, *p* = 0.290; AIE × exercise interaction, F = 8.22, *p* = 0.008; Figure 3A), IL-6 (AIE, F = 14.91, *p* < 0.001; exercise, F = 0.27, *p* = 0.604; AIE × exercise interaction, F = 9.09, *p* = 0.005; Figure 3B) and TNF-α (AIE, F = 8.49, *p* = 0.006; exercise, F = 6.14, *p* = 0.019; AIE × exercise interaction, F = 11.81, *p* = 0.002; Figure 3C) in the hippocampus. Multiple comparison test showed that the IL-1β (*p* < 0.001, Figure 3A), IL-6 (*p* < 0.001, Figure 3B) and TNF-α (*p* < 0.001, Figure 3C) levels were significantly increased in the AS rats than those in the SS rats. However, the IL-1β (*p* = 0.010, Figure 3A), IL-6 (*p* = 0.018, Figure 3B) and TNF-α (*p* < 0.001, Figure 3C) levels were significantly decreased in the AE rats than those in the AS rats. 

A two-way ANOVA showed that AIE and exercise had significant effects on the protein level of IL-10 (AIE, F = 25.17, *p* < 0.001; exercise, F = 0.90, *p* = 0.355; AIE × exercise interaction, F = 7.45, *p* = 0.013; Figure 3D) in the hippocampus. The multiple comparison test showed that the IL-10 level was significantly decreased in the AS rats than those in the SS rats (*p* < 0.001, Figure 3D). Meanwhile, exercise increased the IL-10 level in the hippocampus in the AIE rats (*p* = 0.018, Figure 3D). 

Taken together, these findings suggested that the 8-week treadmill exercise intervention attenuated the neuroinflammation in the hippocampus in AIE rats by reducing the levels of proinflammatory factors and elevating the level of the anti-inflammatory factor.

### 2.3. Treadmill Exercise Decreased AIE-Induced Excessive Activation of Microglia in the Hippocampus of Young Adult Rats

Microglia is one of the key mediators of neuroimmune activation in the central nervous system (CNS). Excessive activation of microglia can release harmful inflammatory factors and exacerbate inflammatory responses. To investigate whether AIE increases the activation of microglia in the hippocampus and whether treadmill exercise affects the activation of microglia in the hippocampus of AIE rats, we detected the number and morphological index of microglia cells, using ionized calcium binding adapter molecule 1 (Iba-1) as the maker of microglia. We also detected the expression of activated microglia marker CD68 in the hippocampus.

**Figure 4:**
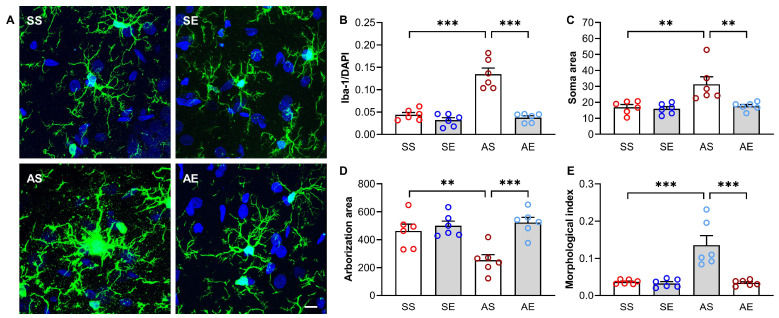
Treadmill exercise decreased the AIE-induced excessive activation of microglia in the hippocampus of young adult rats. (**A**) The representative microscope imaging of Iba-1^+^ cells in the hippocampus of rats in each group. Green, Iba-1^+^ cells. Blue, DAPI. Scale bar = 10 μm. (**B**) The Iba-1/DAPI in the hippocampus of rats in each group. The Iba-1/DAPI was increased in the AS rats compared to the SS rats. The increased Iba-1/DAPI was prevented by treadmill exercise training (*** *p*< 0.001, *n* = 6). (**C**) The soma area of Iba-1^+^ cells in the hippocampus of rats in each group. The soma area of Iba-1^+^ cells was increased in the AS rats compared to the SS rats. The increased soma area of Iba-1^+^ cells was prevented by treadmill exercise training (** *p*< 0.01, *n* = 6). (**D**) The arborization area of Iba-1^+^ cells in the hippocampus of rats in each group. The arborization area of Iba-1^+^ cells was decreased in the AS rats compared to the SS rats. The decreased arborization area of Iba-1^+^ cells was prevented by treadmill exercise training (** *p* < 0.01, *** *p* < 0.001, *n* = 6). (**E**) The morphological index of Iba-1^+^ cells in the hippocampus of rats in each group. The morphological index of Iba-1^+^ cells was increased in the AS rats compared to the SS rats. The increased morphological index of Iba-1^+^ cells was prevented by treadmill exercise training (*** *p* < 0.001, *n* = 6).

The Iba-1 positive staining cells are shown in Figure 4A. A two-way ANOVA showed that there were significant effects of AIE and exercise on the Iba-1/DAPI (AIE, F = 36.52, *p* < 0.001; exercise, F = 46.50, *p* < 0.001; AIE × exercise interaction, F = 28.25, *p* < 0.001; Figure 4B) in the hippocampus. The multiple comparison test showed that the Iba-1/DAPI was significantly increased in the AS rats than those in the SS rats (*p* < 0.001, Figure 4B). Meanwhile, exercise decreased the Iba-1/DAPI in the hippocampus in the AIE rats (*p* < 0.001, Figure 4B). 

A two-way ANOVA showed that there were significant effects of AIE and exercise on the soma area of Iba-1^+^ cells (AIE, F = 9.21, *p* = 0.007; exercise, F = 7.82, *p* = 0.011; AIE × exercise interaction, F = 5.87, *p* = 0.025; Figure 4C), the arborization area of Iba-1^+^ cells (AIE, F = 5.40, *p* = 0.031; exercise, F = 14.68, *p* = 0.001; AIE × exercise interaction, F = 8.43, *p* = 0.009; Figure 4D) and the morphological index of Iba-1^+^ cells (AIE, F = 14.95, *p* < 0.001; exercise, F = 16.54, *p* <0.001; AIE × exercise interaction, F = 14.20, *p* = 0.001; Figure 4E) in the hippocampus. The multiple comparison test showed that the soma area (*p* = 0.001, Figure 4C) and morphological index (*p* < 0.001, Figure 4E) of Iba-1^+^ cells were significantly increased and the arborization area of Iba-1^+^ cells (*p* = 0.002, Figure 4D) was significantly decreased in AS rats compared to those in the SS rats. Meanwhile, exercise decreased the soma area (*p* = 0.002, Figure 4C) and morphological index (*p* < 0.001, Figure 4E) of Iba-1^+^ cells and increased the arborization area of Iba-1^+^ cells (*p* < 0.001, Figure 4D) in the AIE rats.

**Figure 5:**
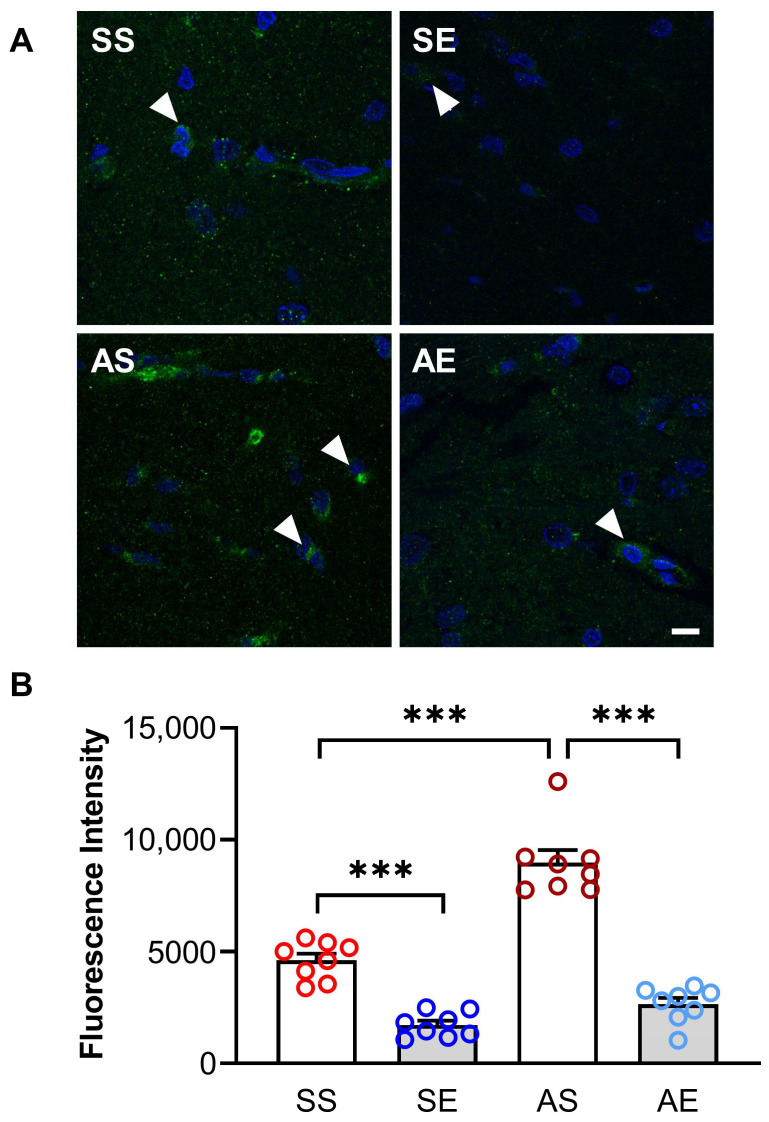
Treadmill exercise decreased the AIE-induced increase in CD68 in the hippocampus of young adult rats. (**A**) Representative microscope imaging of CD68 immunofluorescence staining in the hippocampus of rats in each group. The positive staining of CD68 is marked by white arrowheads. Scale bar = 10 μm. (**B**) The fluorescence intensity of CD68 in the hippocampus of rats in each group. The expression of CD68 was decreased in the SE rats compared to the SS rats. The expression of CD68 was increased in the AS rats compared to the SS rats. The increased expression of CD68 in AIE rats was prevented by treadmill exercise training (*** *p* < 0.001, *n* = 8).

We then examined the expression of CD68 in the hippocampus of rats. The CD68 positive stainings are shown in Figure 5A. A two-way ANOVA showed that there were significant effects of AIE and exercise on the expression of CD68 (AIE, F = 54.22, *p* < 0.001; exercise, F = 163.94, *p* < 0.001; AIE × exercise interaction, F = 22.90, *p* < 0.001; Figure 5B) in the hippocampus. The multiple comparison test showed that the expression of CD68 was significantly increased in the AS rats compared to that in the SS rats (*p* < 0.001, Figure 5B). Meanwhile, exercise decreased the expression of CD68 in the AIE rats (*p* < 0.001, Figure 5B).

Taken together, these findings suggested that the 8-week treadmill exercise intervention reduced the excessive activation of microglia cells in the hippocampus in AIE rats.

### 2.4. Effects of Treadmill Exercise on the Protein Levels of CB1R, CB2R and MAGL in the Hippocampus of Young Adult Rats

**Figure 6:**
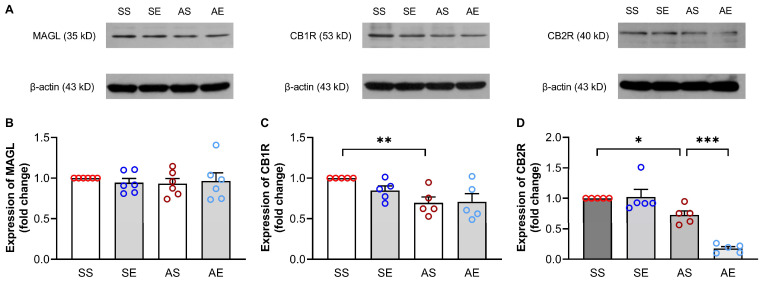
Effects of treadmill exercise on the expression of CB1R, CB2R and MAGL in the hippocampus of young adult rats. (**A**) Representative Western blots for MAGL, CB1R and CB2R in the hippocampus of rats in each group. (**B**) Summarized data show that there were no significant differences in the expression of MAGL in the hippocampus (*p* > 0.05, *n* = 6). (**C**) Summarized data show that the expression of CB1R was decreased in the AS rats compared to the SS rats. Treadmill exercise training had no significant effects on the expression of CB1R in the hippocampus of AIE rats (** *p* < 0.01, *n* = 5). (**D**) Summarized data show that the expression of CB2R was decreased in the AS rats compared to the SS rats. The expression of CB2R was decreased in the AE rats compared to the AS rats (* *p* < 0.05, *** *p* < 0.001, *n* = 5).

It has been shown that the ECS plays a significant role in mediating AIE-induced neuroinflammation. To explore the potential mechanisms of exercise-induced reductions in the excessive activation of microglia cells and neuroinflammation in the hippocampus of AIE rats, we further examined the expressions of MAGL, CB1R, and CB2R in the hippocampus of rats. The representative positive bands for MAGL, CB1R and CB2R are shown in Figure 6A. For the expression of MAGL, a two-way ANOVA showed that there were no significant effects of AIE and exercise on the expression of MAGL (AIE, F = 0.14, *p* = 0.717; exercise, F = 0.04, *p* = 0.852; AIE × exercise interaction, F = 0.45, *p* = 0.511; Figure 6B).

For the expression of CB1R, a two-way ANOVA showed that there was a significant main effect of AIE on the expression of CB1R (F = 10.74, *p* = 0.005, Figure 6C), there was no significant main effect of exercise on the expression of CB1R (F = 1.05, *p* = 0.320, Figure 6C), and there was not a significant interaction between AIE and exercise on the expression of CB1R (F = 1.45, *p* = 0.245, Figure 6C). The multiple comparison test showed that the expression of CB1R was significantly decreased in the AS rats than that in the SS rats (*p* = 0.006, Figure 6C). However, there was no significant effect of exercise on the expression CB1R in the hippocampus of AIE rats (*p* = 0.901, Figure 6C). 

For the expression of CB2R, a two-way ANOVA showed that there were significant effects of AIE and exercise on the expression of CB2R (AIE, F = 61.24, *p* < 0.001; exercise, F = 13.59, *p* = 0.002; AIE × exercise interaction, F = 16.13, *p* < 0.001; Figure 6D). The multiple comparison test showed that the expression of CB2R was significantly decreased in the AS rats than those in the SS rats (*p* = 0.016, Figure 6D). Meanwhile, exercise decreased the expression of CB2R in the hippocampus of AIE rats (*p* < 0.001, Figure 6D).

Taken together, these findings suggest that the protein levels of CB1R and CB2R in the hippocampus changed in AIE rats. The 8-week treadmill exercise intervention modulated the expressions of CB2R in the hippocampus of AIE rats.

## 3. Discussion

In this study, we demonstrated that the AIE rats exhibited impaired cognitive performance, and that the treadmill exercise intervention attenuated the impairment in cognitive performance in AIE rats. The treadmill exercise intervention induced an increase in the level of anti-inflammatory factor, decreases in the level of proinflammatory factors, the activation of microglial cell and the expression of CB2R in the hippocampus of AIE rats. These results suggest that exercise might serve as an effective non-pharmacological intervention to attenuate the AIE-induced cognitive dysfunction.

In this study, the working memory and reference memory were assessed using a right-arm land-based radial arm maze. We found that the working memory error, reference memory error and total time needed to finish each repetition of the maze test were significantly increased in the AIE rats compared to the SS group. The results clearly indicate that cognitive function in AIE rats was impaired compared to the control mice. Consistent with our results, previous studies have shown that heavy alcohol exposure in adolescence could lead to a deficit in adult cognitive performance, reflected by other behavioral tests, including Y-maze [31], Morris water maze [32,33], radial arm water maze, Hebb-Williams maze and tube dominance test [34]. Moreover, the 8-week treadmill exercise intervention significantly decreased the number of working memory errors on day 2 and decreased the time needed to finish the test on day 1, day 2, day 3 and day 4 of the acquisition session in the eight-arm radial maze test. The results confirm that the decline in cognitive performance induced by heavy alcohol exposure in adolescence could be reversed by regular aerobic exercise. These results seem to be consistent with other research which found wheel running and survival-enhancing environmental complexity in adolescence-attenuated alcohol exposure induced deficits in hippocampus-dependent learning and memory, including trace eyeblink conditioning and conditioned fear to the context [24]. However, some inconsistent results of no rescue in cognitive performance after regular aerobic exercise has been found in AIE models [31,35]. Inconsistent results may be due to many different factors such as AIE models, exercise types, exercise intensity and duration and stages of abuse process. Furthermore, the cognitive impairment in AIE rats was different from that in AD animals [36]. The degree of cognitive impairment varies between patients or animal models with different neuropathological stimulation. The results suggest that AIE-induced neuropathological stimulation is much milder than the pathological process during late-life dementia.

We explored the possible mechanisms that might contribute to the 8-week treadmill exercise intervention-induced improvement in cognitive performance in the young adult rats of AIE models. It is believed that inflammatory responses within the hippocampus lead to the dysfunction of learning and memory. Inflammatory responses in the brain are regulated by pro-inflammatory factors and anti-inflammatory factors in opposite manners. Therefore, we tested the concentrations of pro-inflammatory factors IL-1β, IL-6, TNF-α and anti-inflammatory factor IL-10 in the hippocampus of rats in each group. We found that the 8-week treadmill exercise intervention led to an increase in the level of anti-inflammatory cytokine IL-10 in the hippocampus of AIE rats during early adulthood. Meanwhile, the 8-week treadmill exercise decreased the level of pro-inflammatory cytokines, including IL-1β, IL-6 and TNF-α. Previous studies have shown that neuroinflammation was upregulated in the AIE models and were reversed by exercise intervention [16]. Consistent with these studies, we found that the treadmill exercise intervention attenuated the AIE-induced neuroinflammation in the hippocampus. Microglia cells overactivation is thought to underlie the neuroinflammation. In previous studies, the excessive activation of microglia cells was observed in the hippocampus and dorsal raphe nucleus of AIE models [21,37]. Therefore, normalizing microglial activation and inhibiting microglia-induced neuroinflammation in the hippocampus could be helpful to prevent and treat the AIE-induced dysfunction of cognitive performance. We extended these findings by showing that exercise led to a reduction in the activation of microglia cells in the hippocampus of AIE rats. These findings suggest that exercise-induced reduction in the activation of microglia cell contributes to the attenuation of neuroinflammation in the hippocampus, which leads to the regulation of synaptic transmission efficacy and the improvement of the decline in the cognitive performance of AIE rats [29].

A number of studies have demonstrated that endocannabinoids have powerful anti-inflammatory and immunosuppressive properties, and the activation of cannabinoid receptors by endocannabinoids reduces the production of pro-inflammatory factors and increases the secretion of anti-inflammatory factors [30,38,39,40]. MAGL is the primary enzyme responsible for 2-arachidonoylglycerol (2-AG). The expression and activity of MAGL in the hippocampus were changed in some animal models with cognitive impairment [29,41,42]. Thus, we detected the protein level of MAGL in the hippocampus. We found that AIE did not change the expression of MAGL in the hippocampus. This is inconsistent with previous studies reporting that chronic exposure to alcohol during adolescence increased the expression of MAGL in the hippocampus [8]. Inconsistent results may be due to differences in the AIE paradigm (drinking vs. intragastric administration) and differences in the withdrawal duration. Furthermore, we found that 8 weeks of exercise intervention had no effect of the expression of MAGL in the hippocampus of AIE rats. This suggests that MAGL might not be involved in the exercise-induced improvement in cognitive performance. CB1R is considered to be the most abundant G-protein coupled receptor within the brain being expressed in axon terminals and glia cells. In previous studies, CB1R was observed to play a key role in alcohol-induced brain damage. The co-exposure of alcohol and the potent CB1R agonist enhances alcohol-induced brain damage [30]. AIE disrupts a form of excitatory long-term depression that is dependent on CB1R [8]. In the present study, we found that AIE decreased the expression of CB1R in the hippocampus of AIE rats. The result is in line with previous research, suggesting that the decreased protein level of CB1R in the hippocampus might be the potential mechanism of AIE-induced impairments in synaptic plasticity and cognitive performance [8]. Meanwhile, we found that the treadmill exercise intervention had no effect on the protein level of CB1R in the hippocampus of AIE rats. There are three possibilities that may explain this phenomenon. First, CB1R might not be involved in the exercise-induced protection of brain damage in AIE rats. Second, the phenolic endocannabinoids may exert its effects directly as antioxidant chemicals by reducing free radical damage via a non-CB1R pathway [43]. Third, it has been shown that the stimulation of CB1R could diminish the release of proinflammatory factors, iNOS and ROS via NF-κB pathway inhibition and protect neurons from excitotoxicity [44,45,46]. We therefore speculated that treadmill exercise intervention increased the protein level of CB1R in the axon terminals and decreased the CB1R expression in the glia cells. Thus, the total protein level of CB1R was not changed in the hippocampus of AIE rats after treadmill exercise intervention. CB2R is expressed in the microglia cells and involved in the modulation of neuroimmune responses. Previous studies have shown that the expression of CB2R was drastically increased in neurodegenerative disorders or after brain damage [47,48,49]. In the present study, we found that AIE decreased the expression of CB2R in the hippocampus of AIE rats. The result was inconsistent with results observed in neurodegenerative disorders or brain damage [47,48,49]. Compared with neurodegenerative disorders or brain damage, the range of alcohol-induced neuroinflammatory responses is probably less profound and so, its effects on the level of CB2R might be different. In addition, the activation of CB2R is related to decreases in proinflammatory factors [50]. Thus, the decrease in CB2R in the AIE rats might lead to the neuroinflammation in the hippocampus. Interestingly, we found that treadmill exercise intervention further decreased the expression of CB2R in the hippocampus. There are three possibilities that may explain this phenomenon. First, due to its microglia localization, we speculate that the decrease in CB2R in the AIE rats after treadmill exercise intervention might be attributed to the decrease in the number of Iba-1 positive cells. Second, microglia cells also appear to express cannabinoid-activated receptors that are not CB1R and CB2R [30]. This suggests that the endocannabinoid-mediated regulation of microglia cell activation and neuroinflammation might be occurring via non-CB2R pathways. Third, a meta-analysis showed that effects of chronic exercise on the endocannabinoid system were inconsistent. The substantial heterogeneity might depend on the exercise intensity, physical fitness, timing of measurement, and/or fasted state [51]. Thus, the role of ECS in the occurrence and development of alcohol-induced brain disorders is complex. Additionally, there are mixed results on the effects of exercise intervention on the endocannabinoid system in neuropsychiatric disorders. More studies focusing on the effects of alcohol and exercise intervention on the central ECS would provide clarification. 

## 4. Materials and Methods

### 4.1. Animals

Forty male Wistar rats at P21 were acquired from Vital River Laboratory Animal Technology Co. Ltd., Beijing, China. All animals were maintained under temperature control (22 ± 2 °C) and 12 h light–dark cycle (lights on between 06:00 and 18:00) with ad libitum access to food and water. All animal treatments were in accordance with protocols approved by the ethical committee of Beijing Sport University (2020118A).

### 4.2. Adolescent Intermittent Ethanol (AIE) Paradigm

The AIE paradigm referred to the previous research [22]. On P28, all rats were randomly assigned into four groups (*n* = 10 in each group): saline sedentary (SS) group, saline exercise (SE) group, AIE sedentary (AS) group and AIE exercise (AE) group. From P28 to P55, rats in AS and AE groups received a single daily intragastric (i.g.) administration of ethanol (5.0 g/kg, 20% ethanol *w*/*v*) on a two-day on/two-day off schedule. Rats in SS and SE groups received comparable volumes of saline. 

### 4.3. Treadmill Exercise Protocol

The treadmill exercise protocol was based on the previous study [27]. On P25, all animals were introduced to treadmill training on 3 consecutive days (first day, 10 m/min on a 0° slope for 30 min; second day, 15 m/min on a 0° slope for 30 min; third day, 15 m/min on a 0° slope for 60 min). Then, the rats in the SE and AE groups were subjected to a treadmill exercise protocol: 15 m/min on a 0° slope for 10 min, then 18 m/min on a 0° slope for 50 min, 60 min/day, 5 days/week, 8 weeks (P28–P83). According to the previous results [52,53], the running protocol used in this study is a moderate exercise intensity, which is equivalent to 60–65% maximal oxygen uptake (VO_2_max). The SS group and AS group rats were left on the treadmill machine without running for the same period as the exercise group. The animals were used for the behavior test (eight-arm radial maze test) and then sacrificed 24 h after the last exercise to avoid the metabolic effects of the final run.

### 4.4. Eight-Arm Radial Maze Test

The eight-arm radial maze test was performed based on published procedures with minor modifications [36]. In order to increase the motivation for food (chocolate) reaching, dietary restrictions were performed on the rats three days before the test to reach the 80–85% of the initial body weight and maintained under this body weight during the whole experimental process. The radial maze test consisted of two sessions: the adaptation session and the acquisition session. The rats were firstly introduced to the adaption session on three consecutive days. In this session, the rats familiarized the maze environment, during which each rat was allowed to explore and consume food freely for 10 min in each arm of the maze.

During the ten consecutive days (ten trials) of the acquisition session, food was placed in four randomly selected arms. In each trial, the rat was placed in the central platform. Then, the rat was allowed to explore the maze freely. After the rat had visited all four baited arms, the trial finished. Otherwise, the rat was given a maximum of 10 min in each trial. During the acquisition session, the working memory error, the reference memory error and the finish time were recorded. The working memory error was defined as the number of entries into a previously visited baited arm. The reference memory error was defined as the number of entries into a non-baited arm. The finish time was defined as the time needed to complete each test trial. For a specific rat, the food was placed in the same four arms during the ten consecutive trials. The test and data analysis were performed using JLBehv-8ARMM (Shanghai Jiliang Company, Shanghai, China). 

### 4.5. Enzyme-Linked Immunosorbent Assay (ELISA)

The protein levels of interleukin-1 beta (IL-1β), interleukin-6 (IL-6), interleukin-10 (IL-10) and tumor necrosis factor-α (TNF-α) in the hippocampus were determined using ELISA kits. The ELISA was performed according to the manufacturer’s instructions. Briefly, the hippocampus was collected and homogenized in ice-cold RIPA buffer (89900, Pierce, Waltham, MA, USA). The lysate was centrifugated and the supernatant was collected. The total protein concentrate was measured using the BCA method (23227, Pierce, Waltham, MA, USA). The IL-1β, IL-6, IL-10 and TNF-α protein levels were determined by Rat IL-1β ELISA kit (ab100768, Abcam, Boston, MA, USA), Rat IL-6 ELISA kit (ab100772, Abcam, Boston, MA, USA), Rat TNF-α ELISA kit (ab46070, Abcam, Boston, MA, USA) and Rat IL-10 ELISA kit (ab100765, Abcam, Boston, MA, USA), respectively. The optical density was measured by a microplate reader (xMark^TM^, Bio-Rad, Hercules, CA, USA). Finally, the protein levels of inflammatory factors were conducted by normalizing them to total protein levels. 

### 4.6. Immunofluorescent Staining

The immunofluorescent staining was based on the previous study [54]. Rats were anesthetized by isoflurane inhalation. Transcardially, rats were perfused with cold 0.1 M phosphate-buffered saline (PBS) and 4% paraformaldehyde. The whole brain was then removed and immersed in the same fixative solution at 4 °C overnight. After the post-fixation, the brain was dehydrated in 20% and 30% sucrose overnight at 4 °C, snap frozen in liquid nitrogen coated with OCT and stored at −80 °C. Coronal sections (40 μm) were cut on a cryostat (CM3050S, Leica, Nussloch, Germany) and placed onto adhesion slides. Sections were air-dried at room temperature for 2 h and washed in PBS. Then, the sections were blocked with 10% BSA and goat serum for 30 min at room temperature. After blocking, the sections were incubated with Iba-1 (a specific marker for microglia, ab178847, Abcam, Boston, MA, USA) or CD68 (a marker for activated microglia, ab125212, Abcam, Boston, MA, USA) primary antibodies at 4 °C for 48 h. On the third day, sections were washed in PBS and incubated with secondary antibodies (Alexa Fluor 488-Conjugated Goat anti-Rabbit IgG, A-11008, Invitrogen, Shanghai, China) for 60 min in the dark at room temperature. After washing, the sections were covered with antifade solution. The sections were visualized using the Leica SP8 confocal microscope. Image Pro Plus (Media Cybernetics, Silver Spring, MD, USA) was used for image analysis. For the Iba-1 positive cells, the soma area, arborization area and morphological index were detected. The soma area was determined by drawing a line around the cell body, using the freehand selection tool. The arborization area was quantified by measuring the area circumscribed by the distal ends of each process, using the polygon selection tool. The morphological index (the extent of microglia ramification) is the ratio of soma area to arborization area. For the CD68 positive staining, the fluorescence intensity was detected. Micrographs from at least 6 randomly selected areas were analyzed using the same reference position of the section.

### 4.7. Western Blots

Rats were anesthetized by isoflurane and decapitated. The hippocampus was collected, frozen in liquid nitrogen and stored at −80 °C. Tissues were lysed in ice-cold RIPA lysis buffer (89900, Pierce, Waltham, MA, USA) supplemented with protease and phosphatase inhibitor cocktail (Roche, Indianapolis, IN, USA). The lysates were centrifuged at 13,000× *g* for 30 min at 4 °C to collect the supernatants. The total protein concentration was measured using the BCA method (23227, Pierce, Waltham, MA, USA). Samples were diluted using sample buffer and heated at 100 °C for 15 min. Equal amounts of total protein were loaded into a 12% polyacrylamide gel (20 μg/well) for electrophoretic separation and subsequently transferred onto a polyvinylidene difluoride (PVDF) membrane. After blocking in the 10% BSA at room temperature for 2 h, the membranes were incubated in the primary antibodies (Rabbit polyclonal to Cannabinoid Receptor I, ab23703, Abcam, Boston, MA, USA; Rabbit polyclonal to Cannabinoid Receptor II, ab3561, Abcam, Boston, MA, USA; Rabbit polyclonal to Monoacylglycerol Lipase, ab24701, Abcam, Boston, MA, USA) overnight at 4 °C. The next day, the membranes were washed and incubated with the secondary antibodies (HRP-conjugated Affinipure Goat Anti-Rabbit IgG, SA00001-2, Proteintech Group, Rosemont, IL, USA), followed by washes in TBST. Protein levels were detected with chemiluminescent reagents (SuperSignal West Pico Chemiluminescent Substrate, 34580, Pierce, Waltham, MA, USA). The intensity of the special protein band was determined by Image J. To ensure equal total protein loading across the samples, β-actin (YM3028, Immunoway, Plano, TX, USA) served as an internal control. All Western blots were repeated 5–6 times.

### 4.8. Statistical Analysis

Results are presented as mean ± stand error (SEM). Average values of working memory error, reference memory error and time needed to complete each trial were calculated for each individual group on each day of the ten consecutive days. Repeated-measures analysis of variance (ANOVA) was performed to compare the differences in measurements between different days of the same group, between different groups on the same days and the interactions between time and groups. Statistical analysis was conducted using two-way analysis of variance (ANOVA) followed by Tukey post hoc multiple comparison test for other results. A *p* value of less than 0.05 (*p* < 0.05) was considered statistically significant. The F value is the ratio of mean squares between the groups to the mean squares of errors. The statistical analysis was performed using Sigmaplot 14.0 (Systat Software, San Jose, CA, USA).

## 5. Conclusions

AIE-induced neuroinflammation and the excessive activation of the hippocampus microglial cells can be attenuated by treadmill exercise, which might be one of the exercise-induced neural mechanisms protecting against adolescent alcohol abuse. 

## Data Availability

The datasets used in the analyses described in this study are available from the corresponding author on reasonable request.
